# The young adult patient presenting with oligodontia: diagnostic and management strategies

**DOI:** 10.1186/1746-160X-8-S1-I6

**Published:** 2012-05-25

**Authors:** Clark M Stanford

**Affiliations:** 1 Department of Prosthodontics, University of Iowa, USA

## 

Patients with various forms of ectodermal dysplasia often present with oligodontia, aplastic dento-alveolar ridges, malformed teeth and hyposalivation. Management and support follow a continuum from early childhood to adulthood. In addition, management strategies impact on the patient’s perceived quality of life (QOL) as well as on the outcomes of care. The role of the Prosthodontics team is to diagnose, educate and provide care plans that address the range of issues concerning the young adult needing tooth replacement therapy. These often involve ceramic restorations, oral implants and fixed and removable prostheses. Ultimately, the diagnostic phase is critical and requires an interdisciplinary care team leading to rational care plans. Long-term data with regard to oral implant outcomes, complex reconstructions and the impact on QOL will be discussed. There are ranges of treatment options with different advantages and challenges. The young adult with ectodermal dysplasia therefore needs to understand the critical points of assessment, the process of informed consent, the individual care plan, and the possible outcomes of care when electing to perform tooth replacement.

